# EEG Channel Selection Using Particle Swarm Optimization for the Classification of Auditory Event-Related Potentials

**DOI:** 10.1155/2014/350270

**Published:** 2014-03-25

**Authors:** Alejandro Gonzalez, Isao Nambu, Haruhide Hokari, Yasuhiro Wada

**Affiliations:** Department of Electrical Engineering, Nagaoka University of Technology, 1603-1 Kamitomioka, Nagaoka, Niigata 940-2188, Japan

## Abstract

Brain-machine interfaces (BMI) rely on the accurate classification of event-related potentials (ERPs) and their performance greatly depends on the appropriate selection of classifier parameters and features from dense-array electroencephalography (EEG) signals. Moreover, in order to achieve a portable and more compact BMI for practical applications, it is also desirable to use a system capable of accurate classification using information from as few EEG channels as possible. In the present work, we propose a method for classifying P300 ERPs using a combination of Fisher Discriminant Analysis (FDA) and a multiobjective hybrid real-binary Particle Swarm Optimization (MHPSO) algorithm. Specifically, the algorithm searches for the set of EEG channels and classifier parameters that simultaneously maximize the classification accuracy and minimize the number of used channels. The performance of the method is assessed through offline analyses on datasets of auditory ERPs from sound discrimination experiments. The proposed method achieved a higher classification accuracy than that achieved by traditional methods while also using fewer channels. It was also found that the number of channels used for classification can be significantly reduced without greatly compromising the classification accuracy.

## 1. Introduction

A brain-machine interface (BMI) is a system that allows a person to control or communicate with a computer or actuator using only brain signals. Since no muscle movements are needed, such systems are particularly useful to assist patients with motor disabilities such as amyotrophic lateral sclerosis or spinal cord injury.

One type of BMI system makes use of the P300 event-related potential (ERP), a neuroelectrical wave pattern that can be measured with electroencephalography (EEG). This is a pattern that carries information about the state of attention of the user and that can be robustly elicited by various types of stimuli through the oddball paradigm. In the oddball paradigm, the user pays attention to a series of incoming stimuli and must focus on the occurrences of a rare, task-relevant target stimulus hidden amongst frequent, irrelevant nontarget stimuli. Only the target stimuli elicit the P300 response when perceived by the user. The nontarget stimuli, on the other hand, are more frequent and are ignored by the user and thus do not elicit the P300 response. Therefore, by presenting to the user a stream of stimuli while measuring the user's brain the activity and then detecting the presence or absence of the P300 ERP, one can determine the intention of the user.

An example of this kind of system is the one proposed and investigated in [1–3]. This system allows a handicapped patient to communicate to a computer the direction of attention by detecting the P300 ERPs elicited with auditory stimuli from virtual-sound sources. By using the virtual sounds as the stimuli in the oddball paradigm, it is possible to estimate the intended direction of the user and thus control a transportation device like an electric wheelchair. The use of virtual sound stimuli allows the construction of more portable BMI systems and, in contrast to visual stimuli, allows the user to dedicate his or her vision to other tasks.

The detection of the P300 component is performed by means of a binary classifier fed with the signal features provided by the EEG signals, and its performance greatly depends on the chosen features. Ideally only the most discriminative features should be used to feed the classifier but, in this case, it is usually not immediately clear which is the channel set that provides the most relevant information. Furthermore, the optimal set might not be equal for every subject. One could attempt to perform an exhaustive test of all possible combinations, but, in the case of dense array measurements, the extremely high number of combinations that exist (2^64^, for a 64-channel array) render this approach intractable. Additionally, in practical applications, a BMI system for transportation purposes like the one described in [3] requires compact equipment and it is thus desirable to use a ERP detection system that employs as few channels as possible.

Stepwise Linear Discriminant Analysis (SWLDA) is one of the most popular classifiers used for the detection of ERPs, being used in numerous reports (see, e.g., [4–7]). This classifier is based on the feature selection performed by the Stepwise Regression algorithm in which the features that contribute the most are selected in a stepwise manner; that is, every feature is sequentially added or removed while measuring the predicting power at each step. However, a drawback of this algorithm is that the selection of the features depends on the order on which they are evaluated, especially when there are high correlations between the features [8] which is precisely the case in EEG measurements. Another algorithm worth mentioning is the one proposed by [9] that achieved the best performance in the BCI Competition III [10]. This algorithm performs a stepwise selection of channels following a classification accuracy maximization criterion, but, like SWLDA, the outcome of the procedure also depends on the order of the evaluation of channels. There are also algorithms that employ methods based on Principal Component Analysis (PCA) to extract spatial features [6, 11, 12], but these typically disregard channel selection and employ all the channels available to identify the most discriminative spatial information.

To address the abovementioned points, we propose an alternative approach in which the channel selection procedure is performed in an automated manner aimed at maximizing the classification accuracy of the system. Specifically, we propose a method for classifying P300 ERPs in which the features and the parameters of the classifier are tuned using a random optimization algorithm and evaluate it using experimental data. The proposed method performs classification using Fisher Discriminant Analysis (FDA) and uses a multiobjective hybrid real-binary Particle Swarm Optimization (MHPSO) algorithm to search for the classifier parameters and EEG channel set that simultaneously maximize the classification accuracy and minimize the number of channels used for classification. The PSO algorithm is multiobjective in the sense that it seeks to optimize a fitness function product of an aggregation of two performance metrics (i.e., the classification accuracy and the number of EEG channels) and hybrid real-binary in the sense that the search space is composed of both real and binary dimensions, necessary to tune the FDA regularization parameter (a real variable) and the addition or removal of EEG channels (a binary variable per channel).

PSO [13] is a relatively new stochastic algorithm for function optimization that has become increasingly popular and has also been applied in various fields [14]. This algorithm, inspired in the motion of fish schools that search for food, moves a swarm of particles around a parameter space searching for a solution that maximizes a certain fitness function. Like other stochastic search algorithms, this is a method that does not rely on the gradient of the function to optimize and instead looks for the best solution in a quasirandom way, being particularly useful in problems where an analytical expression for the optimization function is not available. It is important to remark that the PSO algorithm is not guaranteed to converge towards the global maximum. However, this algorithm has been applied with success in a wide variety of applications [14] and in practice tends to find a suitable solution if not the optimal one.

This text is organized as follows. First, the main components of the proposed algorithms, namely, the FDA and PSO algorithms, and their integration to classify ERP signals are described in Section 2. Then, the data and simulations used to evaluate the algorithm are described in Section 3. Lastly, in Section 4, the simulations results are shown and discussed and conclusions are given in Section 5.

## 2. Methods

*Note on Mathematical Notation*. Throughout this work, scalars are denoted by lowercase italic letters (e.g., *x*), vectors are written in lowercase, bold letters (e.g., **x**), and matrices are denoted by uppercase bold letters (e.g., **X**). All vectors are column vectors unless stated otherwise.

### 2.1. Particle Swarm Optimization

The objective of the PSO algorithm is to find the best parameters that maximize a given fitness function, and it does so by iteratively moving a swarm of particles around the parameter space according to especial equations. Each particle of the swarm is a particular position in the parameter space and represents a possible solution to the problem. Mathematically, a particle in PSO is a vector in an *N*-dimensional parameter space, and its position **x** and velocity **v** change according to the following equations:

(1) $$\begin{array}{c} x_{i,j}^{t}=x_{i,j}^{t-1}+v_{i,j}^{t}, \end{array}$$

(2) $$\begin{array}{c} v_{i,j}^{t}=wv_{i,j}^{t-1}+c_{1}\eta_{1}\left(p_{i,j}-x_{i,j}^{t-1}\right)+c_{2}\eta_{2}\left(g_{j}-x_{i,j}^{t-1}\right), \end{array}$$

where *x*_*i*,*j*_^*t*^ and *v*_*i*,*j*_^*t*^ are the *j*th components of the *i*th particle's position and velocity, respectively, at iteration *t*. *p*_*i*,*j*_ is the *j*th component of **p**, the best position that the *i*th particle has found so far, and *g*_*j*_ is the *j*th component of **g**, the best position found by the swarm. The effect of **p** and  **g** on the particle's motion is controlled by two constant parameters *c*_1_ and *c*_2_, and two independent random variables *η*_1_ and *η*_2_ uniformly distributed in [0,1]. The particle's motion is also influenced by the velocity at the previous iteration and this effect is controlled by the inertia parameter *w*.

*c*_1_ and *c*_2_ are constants set by the experimenter that determine the balance between the exploitation of a potential solution (movement towards **g**) and the exploration for new solutions (movement towards **p**). At each iteration, **p** and  **g** are updated if positions with better fitness were found. This is the main feature of the PSO algorithm: each particle uses the information of its own history and the swarm's history, together with random perturbations, to search for the global maximum. The PSO algorithm, in an iterative manner, updates the velocity and positions of each particle, moving them around the parameter space, until the best global solution **g** reaches the desired fitness.

In practical applications, the search space is typically constrained to [*X*_*j*_^min⁡^, *X*_*j*_^max⁡^] along each dimension *j* in order to limit the search space to feasible values. In this work, we enforced an invisible wall condition [15] in which the fitness of the particles that fly out of this region is neither calculated nor updated. Instead, the particles that stray out are expected (but not guaranteed) to eventually fly back into the admissible region. The invisible wall condition has the advantage of being simple to implement and avoiding the unnecessary computations required by the evaluation of unfeasible solutions. Lastly, to avoid the particles flying out of the feasible space too often, the velocity components are limited to a maximum value *V*_*j*_^max⁡^ such that

(3) $$\begin{array}{c} \left|v_{i,j}^{t}\right|<V_{j}^{\max}. \end{array}$$

*Hybrid Binary-Real PSO*. In order to perform channel selection, in this work, we adopted a hybrid binary-real PSO algorithm that, in addition to real-valued variables, allows the optimization of variables that can take only two discrete values. In this case, the additional binary components are updated as shown below [15–17]:

(4) $$\begin{array}{c} x_{i,j}^{t}=\{\begin{array}{cc} 1\; & \text{if}\;r<S\left(v_{i,j}^{t}\right)\\ 0\; & \text{if}\;r\geq S\left(v_{i,j}^{t}\right) \end{array},\;S\left(x\right)=\frac{1}{1+e^{-x}}, \end{array}$$

where *r* is a random number with a uniform distribution in [0,1] and the velocity components *v*_*i*,*j*_^*t*^ are updated in a manner similar to the real case (see (2)). This equation means that, at each iteration, the binary component *x*_*i*,*j*_^*t*^ will be 1 (i.e., the associated channel will be selected) with a probability of *S*(*v*_*i*,*j*_^*t*^) (and 0 with a probability of 1 − *S*(*v*_*i*,*j*_^*t*^)).

### 2.2. Fisher Discriminant Analysis

In this work, a linear binary classifier based on Fisher Discriminant Analysis (FDA) was applied to discriminate between target and nontarget signals. FDA is a machine learning technique proposed by [18] used for data classification. Strictly speaking, FDA is a dimensionality reduction used as a preprocessing step prior to classification and its goal is to find a linear combination of the features that maximizes the separation between the classes' distributions in the reduced space. Classification is then performed in this 1-dimensional space by applying some threshold criteria or by using any classifier trained on the transformed samples. Given a training dataset of *n*-dimensional samples {(**z**_*i*_, *y*_*i*_), *i* = 1,…, *ℓ*} (i.e., each sample has *n* features) were each of the samples belonging to either one of two classes *K*_1_ (positive, e.g., target examples) or *K*_−1_ (negative, e.g., nontarget examples) as indicated by the categorical variable *y*_*i*_ = {+1, −1}, the transformation vector **w** is obtained by maximizing the following function:

(5) $$\begin{array}{c} J\left(w\right)=\frac{{\langle w,m_{1}-m_{-1}\rangle }^{2}}{w^{T}S_{W}w}. \end{array}$$

(〈·, ·〉 denotes the inner product of vectors,) **m**_1_ and **m**_−1_ are the means of the feature vectors of the positive and negative classes, respectively, and **S**_*W*_ is the within-class scatter matrix given by

(6) $$\begin{array}{c} S_{W}=\underset{j\in \;\{1,-1\}}{\sum }\;\underset{z_{i}\in K_{j}}{\sum }\left(z_{i}-m_{j}\right){\left(z_{i}-m_{j}\right)}^{T}, \end{array}$$

with

(7) $$\begin{array}{c} m_{1}=\frac{1}{\ell_{1}}\underset{z_{i}\in K_{1}}{\sum }z_{i},\;\;m_{-1}=\frac{1}{\ell_{-1}}\underset{z_{i}\in K_{-1}}{\sum }z_{i}. \end{array}$$

The maximization of *J*(**w**) therefore yields

(8) $$\begin{array}{c} w={S_{W}}^{-1}\left(m_{1}-m_{-1}\right), \end{array}$$

and the classification of a new, unknown sample, **z**, is performed upon the score

(9) $$\begin{array}{c} s\left(z\right)=\langle w,z\rangle , \end{array}$$

which is the distance of the sample to the hyperplane parameterized by the normal vector **w** and represents a measure of the certainty about the class prediction. Regarding the class prediction, in this work, the class of the sample was determined by

(10) $$\begin{array}{c} \hat{y}=\underset{j\in \left\{1,-1\right\}}{\arg\;\min}\sqrt{\frac{{\left(s\left(z\right)-\mu_{j}\right)}^{2}}{\sigma_{j}^{2}}}, \end{array}$$

where *μ*_*j*_ and *σ*_*j*_ are the mean and variance of the scores of the training samples belonging to class *K*_*j*_ (*j* ∈ {1, −1}) in the transformed space. Equation (10) means that the predicted class corresponds to the class that yields the shortest Mahalanobis distance to the sample.

The key element in the computation of **w** is the scatter matrix **S**_*W*_. **S**_*W*_ is an unbiased estimator of the true, unknown scatter matrix, and it may become imprecise when the number of features is high in comparison to the number of training samples. This is because the number of unknown parameters (the elements of the matrix) is quadratic in the number of features. An imprecise estimation of the within-class scatter matrix results in a degradation of classification performance [5]. To mitigate this effect, regularization is typically applied to the scatter matrix estimation and this is achieved by maximizing a modified target function *J*(**w**) [19]:

(11) $$\begin{array}{c} J\left(w\right)=\frac{{\langle w,m_{1}-m_{-1}\rangle }^{2}}{w^{T}S_{W}w+\lambda {||w||}^{2}}, \end{array}$$

which results in

(12) $$\begin{array}{c} w={\left(S_{W}+\lambda I\right)}^{-1}\left(m_{1}-m_{-1}\right), \end{array}$$

where *λ* is the regularization parameter. It can be seen that for *λ* = 0 the canonical, unregularized FDA is obtained. Thus, it is necessary to appropriately choose the regularization parameter in order to achieve the best performance.

### 2.3. The Proposed Algorithm

#### 2.3.1. Particle Encoding

As was mentioned before, the algorithm uses FDA for classification and a multiobjective hybrid PSO to tune, for each particular user, the channel set and classifier parameters that maximize the classification accuracy using as few channels as possible.

The proposed method employs a hybrid PSO algorithm to search in a space that contains both real and discrete (binary) dimensions in a similar fashion to [15, 17]. These dimensions correspond to the variables that are to be tuned. Thus, each particle is a position in the search space that represents a particular combination of FDA parameters and EEG channels and is a candidate solution for the problem. The channel set and classifier parameters are encoded in a particle **x** as follows:

(13) $$\begin{array}{c} x=\left[\begin{array}{cccc} a & b_{1} & \cdots & b_{64} \end{array}\right], \end{array}$$

where *a* ∈ [−1, 1] and *b*_*j*_ = {0, 1}  ∀*j* denote the real and binary components, respectively. Each binary component (64 in total) *b*_*j*_ encodes whether the temporal features of the corresponding *j*th channel are used for classification (*b*_*j*_ = 1) or not (*b*_*j*_ = 0). Therefore, this encoding results in a search space of 65 dimensions. The real component *a* encodes the FDA regularization parameter *λ*, which is decoded as

(14) $$\begin{array}{c} \lambda =10^{5a}. \end{array}$$

This decoding was chosen because the feasible values for the regularization parameter usually range over several orders of magnitude, and by adopting an exponential representation, the particle can search with small steps for possible solutions over a broader interval.

#### 2.3.2. Fitness Function

As mentioned above, each particle is a candidate solution and represents a possible combination of set of channels and FDA parameters. In this work, we search for the solution that yields the best performance measured in terms of the fitness function *F*:

(15) F(x)=w1f1(x)+w2f2(x)=w1TPPs×TNNs+w2(NCh−n+1NCh),

where *TP* stands for true positives, *TN* stands for true negatives, *P*_*s*_ and *N*_*s*_ are the total number of positive (target) and negative (nontarget) samples, respectively, *N*_Ch_ is the maximum number of channels that can be selected (64 in this work), and *n* is the number of channels in the set specified by the particle. In other words, *F* is a weighted aggregation of the optimization objectives *f*_1_ and *f*_2_: the maximization of the geometric mean between the true positive rate (target accuracy) and the true negative rate (nontarget accuracy) and the minimization of the number of channels. The trade-off between accuracy and number of channels is controlled by the fitness weights *w*_1_ and *w*_2_. Since the ratio between the weights is what actually determines the trade-off, in this work, we chose weights such that *w*_1_ + *w*_2_ = 1. The geometric mean was chosen over the arithmetic mean because it yields an aggressive evaluation in which solutions with low and/or highly unbalanced accuracies are heavily penalized. It is important to notice that the target and nontarget accuracies are not expressed as percentages and thus *f*_1_ varies within [0,1], being 1 the perfect accuracy. *f*_2_, on the other hand, yields higher values for fewer channels and becomes 1 when only one channel is selected. It is important to remark that, although the algorithm attempts to find a solution that yields a perfect classification using only one channel (fitness value equal to 1), such a solution might not exist because the information provided by one channel might not be enough to accurately discriminate the ERP signals.

**Figure 1:**
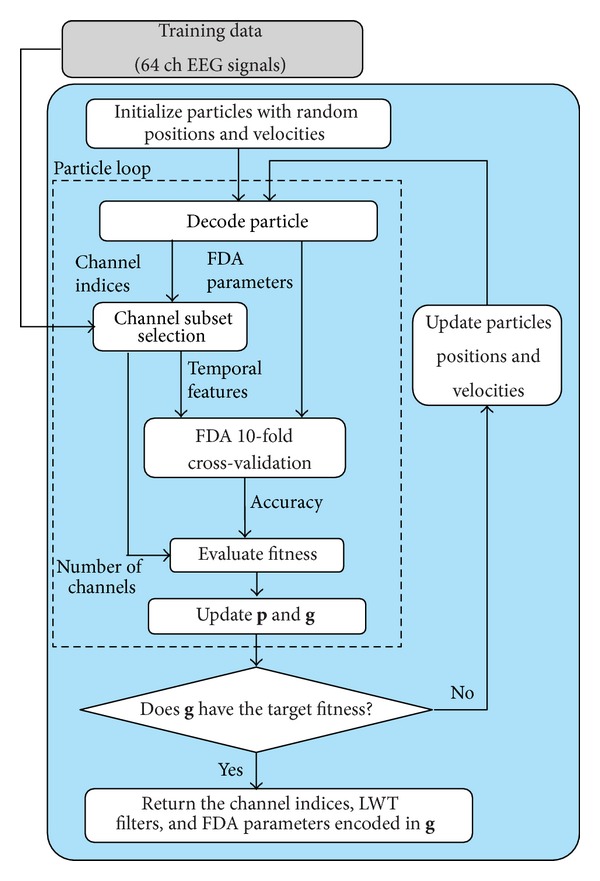
Flowchart of the proposed algorithm.

In the offline analyses, for each particle, a classifier is built using the features and parameters encoded in the particle. Then, the true positive and true negative rates achieved by the classifier are estimated using 10-fold stratified cross-validation on the training data (all folds share the same target to nontarget sample ratio), and the particle fitness is finally calculated using (15). The proposed algorithm's flowchart is shown in Figure 1. A final classifier can then be trained using the best setup found by the algorithm and be used to classify signals in a real-time setting.

## 3. Simulations

### 3.1. Data Set Used in the Study

**Figure 2:**
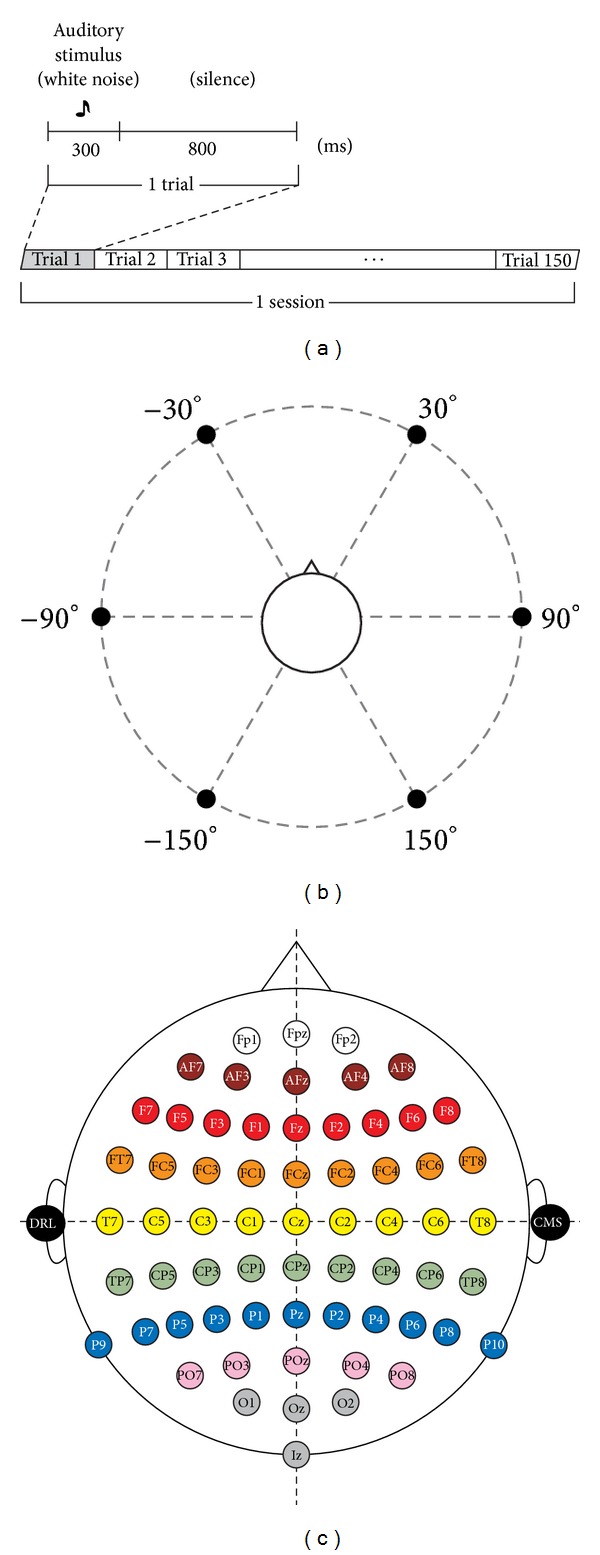
(a) Experimental protocol used to measure the auditory P300 signals. (b) Direction of the virtual sound sources. (c) 64 EEG channel layout used in the experiments. DRL and CMS are the references attached to the earlobes.

The data used in this work corresponds to the data gathered by [1, 2] of sound discrimination experiments that consisted of random presentations of virtual auditory stimuli from 6 directions. During these experiments, the subject had to focus his attention on one of these directions (the target direction) and count every time the stimulus source corresponded to the target direction. The subject had to ignore the stimuli from other directions (the nontarget directions). Each session consisted of 150 trials and each trial consisted of a 300 ms stimulus interval and an 800 ms silence interval. The stimulus was 300 ms of pure white noise. One of the 6 directions was fixed as the target direction throughout a single session and the corresponding stimuli were presented with a probability of 20%. Each direction was measured twice, thus yielding 12 recording sessions and a total of 1800 trials. Thus, about 20% of the samples in the data set correspond to target signals. This protocol is summarized in Figure 2(a) and the directions of the virtual sound sources are shown in Figure 2(b). The neural activity was recorded using a digital electroencephalograph (Active Two, Biosemi, Amsterdam, The Netherlands) with 64 electrodes attached to the subject's scalp using a cap. The electrodes were placed in accordance with the 10–20 system shown in Figure 2(c) and the reference was attached to the earlobes. Twelve healthy men (aged 22–24 years) participated in the experiment.

### 3.2. PSO Algorithm Setup

Preliminary tests were carried out to choose the best PSO parameters among the values suggested in [20, 21]. The chosen values are summarized as follows. All the PSO simulations were performed using 30 particles until a fitness value equal to 1 was achieved or until 100 iterations were exceeded. For the real part, the inertia parameter *w* was varied from 0.9 to 0.4 linearly across 100 iterations and had a constant value equal to 1 for the binary part. Both exploration and exploitation constants were set to *c*_1_ = *c*_2_ = 2. The search space of the only real component was constrained to [−1,1]. The algorithm employed an invisible wall boundary condition and particles that encoded an empty channel set (i.e., all binary components equal to zero) were deemed as out-of-range. The maximum and minimum velocities were set to −0.1 and 0.1, respectively, for the real part, and −6 and 6, respectively, for the binary part. Regarding the particle's initial conditions at the start of the PSO algorithm, the real component was initialized to a random value in [−1,1] and each binary component was randomly initialized to either 0 or 1 with equal probability; thus, on average, each particle began the search process with half of the total channels selected.

### 3.3. Data Preprocessing and Features Used for Classification

Prior to classification, the data was divided in trials, with each trial being the 1100 ms segment of signal starting from −100 ms before the stimulus onset. Then, a zero-phase 3rd order Butterworth band-pass filter with cutoff frequencies of 0.1 Hz and 8 Hz was applied to all the signals across all channels. A baseline correction was performed by subtracting to each trial, at every channel, the mean of the signal in the prestimulus interval [−100,0] ms. Lastly, in order to reduce the number of temporal samples, each trial was downsampled by taking the average of every 10 samples.

The FDA classifier was fed with 25 temporal samples per EEG channel corresponding to the [0,1000] ms trial segment. Thus the number of total features ranges from 25 to 1600 depending on the number of channels selected by the PSO algorithm.

### 3.4. Validation

The performance of the proposed algorithm was assessed by training and testing the algorithm on two disjoint training and test subsets of the original dataset (1800 single-trial samples in total). First, using the training dataset, the algorithm searches for the combination of channels and FDA parameters that maximize the classification accuracy and, when it finishes, it outputs the best configuration that could be found. A final classifier is then trained using the training dataset and the channel and classifier configuration specified by the output of the algorithm. Lastly, the performance of the final classifier is assessed by evaluating its classification accuracy on the test dataset. For the sake of consistency, all the classification accuracies reported in this work are derived from the same criteria used in the fitness function of the MHPSO algorithm, that is, the geometric mean of the target and nontarget accuracies. The subsets were made in such a way that trials of each type (i.e., target and nontarget), each direction (1 to 6), and each session (1 and 2) were present in the subsets with the same proportion as in the original set. Both the training and test sets had approximately 900 samples.

In addition to the single-trial accuracy, in all cases, the averaged-trial accuracy was also assessed. Trial-averaging is a technique usually employed to counteract the high level of noise typically found in EEG signals, and thus improve the classification accuracy. The improved accuracy comes at the cost of a reduced communication speed since several trials must be measured to produce a single estimation. In this work, a *M* averaged-trial was fabricated from the average classification score (using the score defined in (9)) of *M* single-trials of the same class randomly chosen with resampling. A set of averaged-trials is built by repeating this process until a given number of trials are fabricated. The number of samples in the averaged-trial dataset was made equal to the number of samples in the single-trial data set (around 900 samples) with the same ratio of target to nontarget samples. In reality, we made a master list containing the indexes of the single-trials used to make each averaged-trial and used this list in all cases and all subjects to enforce that the averaged-trial datasets were fabricated using exactly the same information and thus ensure a fair comparison between cases. The averaged-trial accuracy was assessed for *M* = 2 … 10.

Lastly, it is important to remark that score averaging was chosen over signal averaging because, if signals are averaged prior to classification, then (1) the number of samples available to train the classifier is reduced, and (2) the P300 component may cancel out if inter-trial jitter is present.

### 3.5. Simulation Cases

For every subject, eight cases were simulated to study the effect of the fitness function weights on the classification accuracy and number of selected channels that are ultimately achieved by the MHPSO algorithm. These cases are listed below.

 Case 1: *w*_1_ = 1.00, *w*_1_ = 0.00.
 Case 2: *w*_1_ = 0.95, *w*_1_ = 0.05.
 Case 3: *w*_1_ = 0.90, *w*_1_ = 0.10.
 Case 4: *w*_1_ = 0.85, *w*_1_ = 0.15.
 Case 5: *w*_1_ = 0.75, *w*_1_ = 0.25.
 Case 6: *w*_1_ = 0.65, *w*_1_ = 0.35.
 Case 7: *w*_1_ = 0.50, *w*_1_ = 0.50.
 Case 8: *w*_1_ = 0.35, *w*_1_ = 0.65.

Additionally, the algorithm was compared to a version that uses all 64 channels without channel selection and only tunes the FDA parameter (Fixed 64), SWLDA, and a combination of spatial PCA and SWLDA (PCA-SWLDA) similar to the one used in [6]. In the latter cases, the SWLDA algorithm was configured with a feature insertion *P* value of 0.1 and a feature removal *P* value of 0.15 as recommended by [7]. In the PCA-SWLDA case, the spatial principal components that accounted for 90% of the variability (the specific number of spatial factors varied between subjects) were used to transform the data prior to classification with SWLDA. In the SWLDA case, there were 1600 features to choose from (25 temporal features per channel) and, in the PCA-SWLDA case, there were between 75 and 200 features available for selection (25 transformed temporal features per principal component).

## 4. Results and Discussion

**Figure 3:**
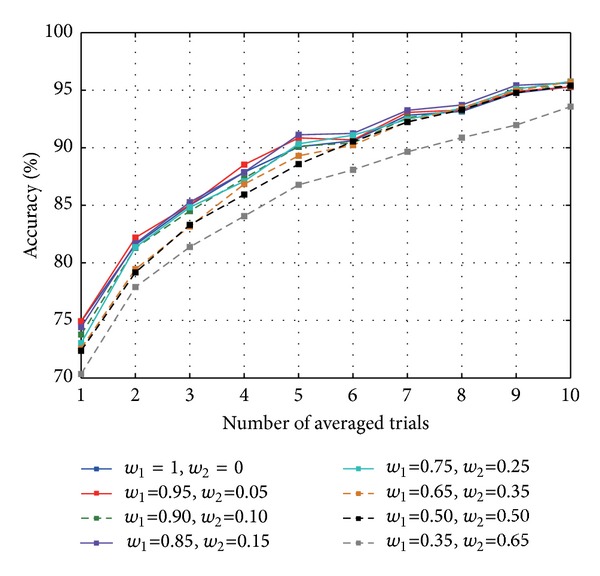
Average classification accuracy of 12 subjects as a function of the number of averaged samples for each case.

The average classification accuracy of 12 subjects as a function of the number of averaged samples for each MHPSO case is shown in Figure 3. From here, first, an improvement of classification with increasing trial averaging can be seen, as is usually expected. A two-way repeated measures analysis of variance (ANOVA) on the classification accuracy (factors: case and number of averaged samples) revealed a statistically significant difference between cases (*F*(7,77) = 3.440, *P* < 0.005) and between the number of averaged samples (*F*(9,99) = 266.93, *P* < 0.001). Since a significant interaction between the case and the number of averaged samples was also found (*F*(63,693) = 2.733, *P* < 0.001), we performed analysis separately for the single-trial and the 10 averaged-trial cases.

**Figure 4:**
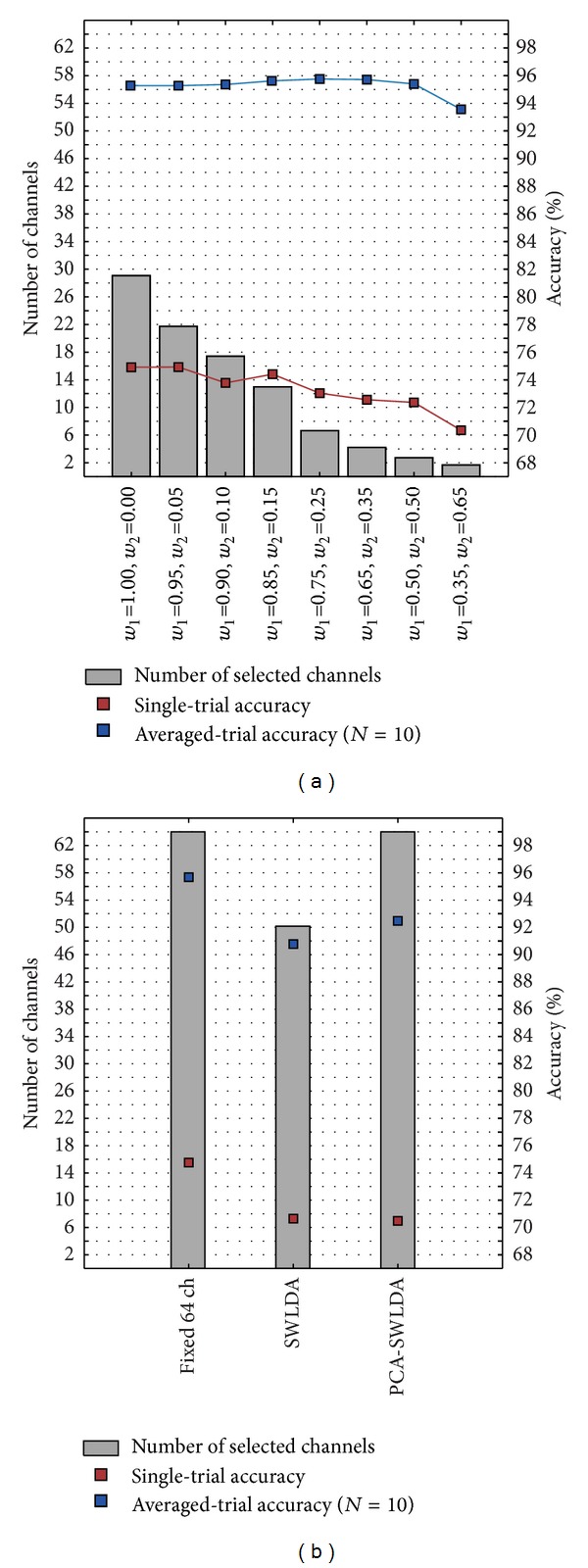
Number of selected channels (gray bars), single-trial (red line), and 10 averaged-trial (blue line) classification accuracy for (a) each MHPSO case and (b) the Fixed 64, SWLDA, and PCA-SWLDA cases.

The number of channels selected and the single-trial and 10 averaged-trial accuracies achieved in each MHPSO case are shown in Figure 4(a). The corresponding results for the Fixed 64, SWLDA, and PCA-SWLDA cases are shown in Figure 4(b). This figure illustrates the effect that fitness weights have on the number of selected channels. A one-way repeated measures ANOVA with Greenhouse-Geisser (GG) correction was conducted on the number of channels (factor: case), revealing a significant difference between the number of channels selected by each case (*ε* = 0.593, *F*(4.15,45.62) = 262.470, *P* < 0.001). A* post hoc* multiple comparison test based on Holm's method revealed a statistically significant difference between all pairs of cases. Higher *w*_2_/*w*_1_ ratios indeed produced a more aggressive channel selection and selected sets with fewer channels.

While greater values of *w*_2_/*w*_1_  yielded fewer selected channels on average, the single-trial and 10 averaged-trial accuracies did not significantly change. A one-way repeated measures ANOVA with GG correction conducted on the single-trial accuracies (factor: case) found a significant difference between cases (*ε* = 0.434, *F*(3.035,33.383) = 8.085, *P* < 0.001), but a* post hoc* Holm test found that these differences were only significant between pairs 1–8 (*P* = 0.027), 2–8 (*P* = 0.027) and 4–8 (*P* = 0.027). A similar analysis performed on the 10 averaged-trial accuracies did not find any significant difference between cases. These results suggest that the number of channels can be reduced without significantly hindering classification accuracy. For example, in Case 7, it can be seen that, with as few as 3 channels, the proposed algorithm could attain a single-trial accuracy slightly slower (around 3%) and an averaged-trial accuracy similar to what would be obtained using the full channel set.

**Figure 5:**
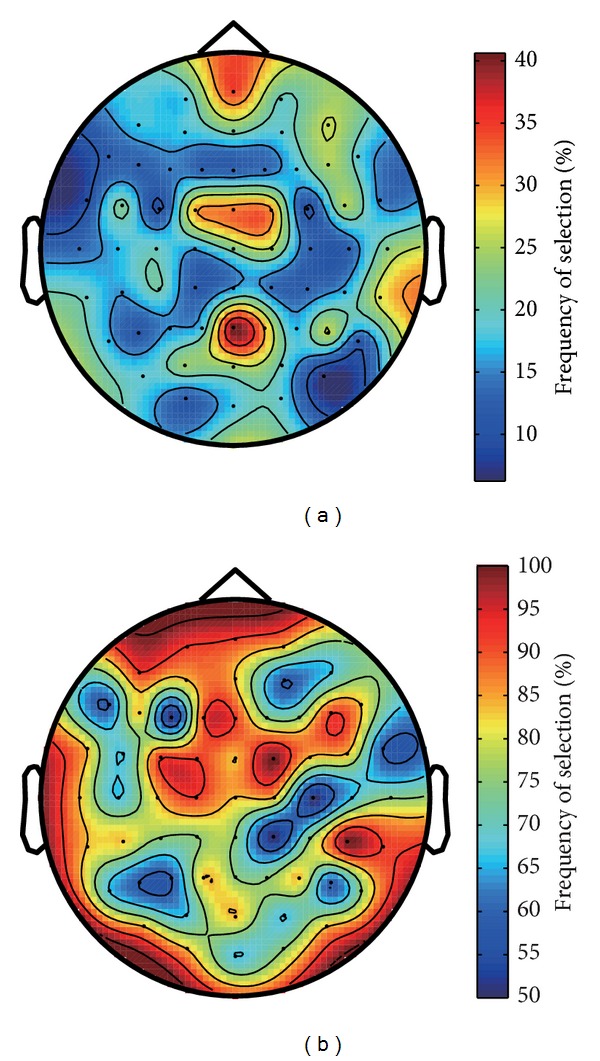
Frequency of selection of channels in (a) the proposed algorithm (across all MHPSO cases and subjects) and (b) SWLDA (across all subjects). Note that the color gradations denote different ranges in each figure.

The number of channels selected by the proposed and SWLDA algorithms were also compared. The frequency with which each channel was selected in both cases is shown in Figure 5. Two observations can be made from this figure. The first observation is that the SWLDA algorithm tended to choose most of the channels in most of the subjects, with the least frequently selected channels being chosen at least half of the time. A one-way repeated measures ANOVA conducted on the number of channels with GG correction (factor: case) showed a significant difference between the channels selected by the proposed method and SWLDA (*ε* = 0.344, *F*(2.755,30.305) = 366.49, *P* < 0.001). All cases were significantly different to SWLDA (*P* < 0.001 for all pairs), confirming that indeed the proposed algorithm adapts sets with fewer channels. We hypothesized that the random initialization of the particles' binary bits gave the proposed algorithm an unfair advantage over other methods, but simulations where all the particle's binary part were initialized to all ones (i.e., all the channels selected) yielded similar results (results omitted for brevity). The second observation is that the proposed algorithm had a tendency to choose channels over the parietal, frontal, and frontal polar regions, as evidenced by the warm-colored spots over channels Pz, FC1, FCz, FC2, and FPz. While the parietal and frontal channels are in line with the spatial behavior of the P300 [22], the FPz channel is presumably being selected by the proposed algorithm to provide a mean of indirect noise reduction in the FDA classifier as suggested by [5, 23].

**Figure 6:**
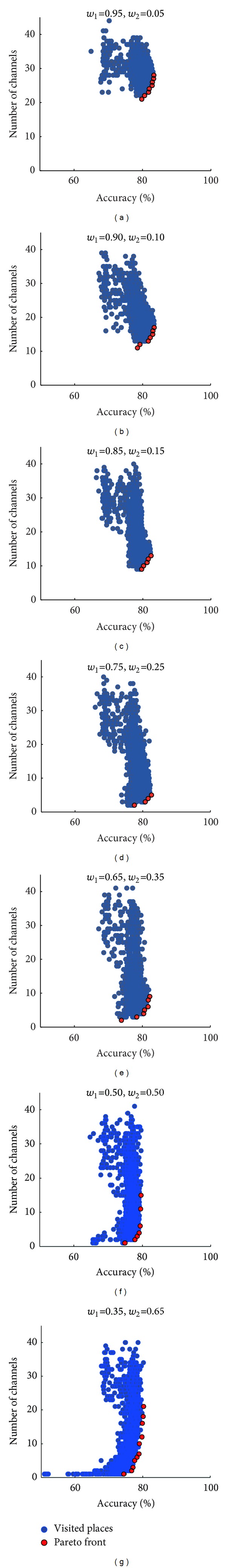
Illustration of the Pareto front found by the algorithm in each MHPSO case for a representative subject. The vertical and horizontal axes denote the number of selected channels and the average classification accuracy, respectively. The blue dots show the fitness of all the positions visited by the swarm. The red dots represent are the positions that belong to the Pareto front. The first MHPSO case was omitted because the Pareto front in this case is meaningless.

Another way to assess the trade-off relationship between the number of channels and the classification accuracy is to study the Pareto front encountered by the particles throughout the course of the optimization process. The Pareto front in this case represents the boundary at which an improvement of classification accuracy necessarily induces an increase of the number of channels or, conversely, an attempt to reduce the number of channels produces a loss of accuracy. An example of the Pareto front found in each case for a representative subject is shown in Figure 6 to illustrate this phenomenon. By looking at Figure 6, it becomes clear that, for increasing values of *w*_2_/*w*_1_, the positions visited by the swarm gradually move towards the bottom and that once an accuracy of about 80% is reached, the Pareto front is encountered and a trade-off between number of channels and accuracy begins. Nevertheless, the existence of positions with equal accuracy but different number of channels that can be seen in any of the cases is in line with the abovementioned results.

**Figure 7:**
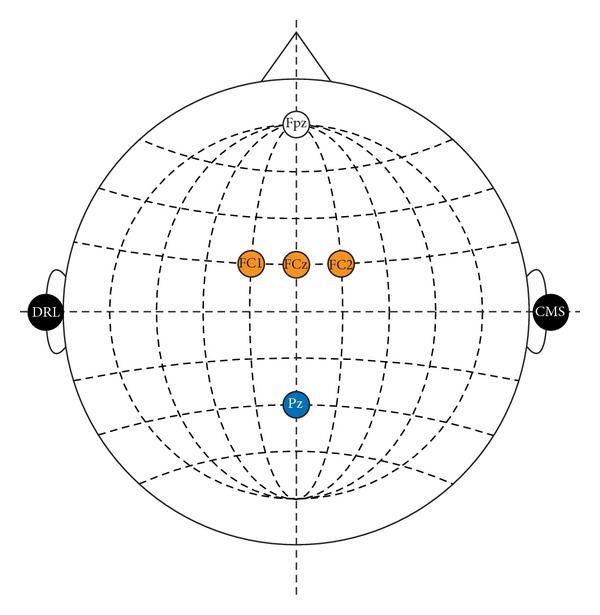
Channel set of 5 fixed channels built from the channels that were most frequently selected by the proposed algorithm.

**Figure 8:**
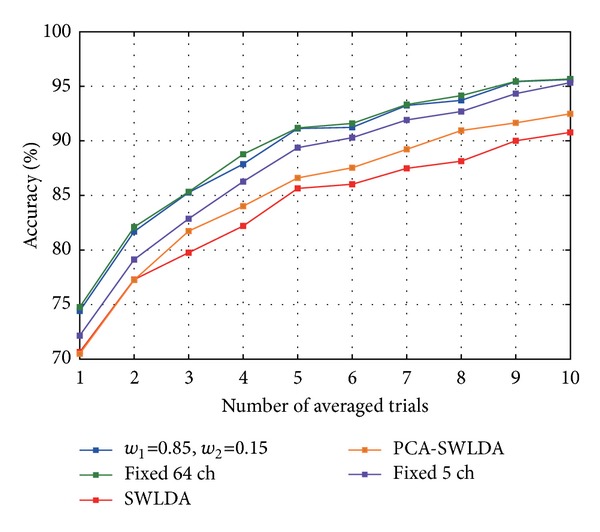
Average classification accuracy across 12 subjects achieved by the Fixed 5 case (purple line). Other colors show the accuracy obtained by the Fixed 64, SWLDA, PCA-SWLDA, and the MHPSO(4) cases.

Lastly, following the results shown in Figure 5(a), it is worth asking if one could use MHPSO to find the most important channels for classification, make a fixed a channel set with a few of those channels, and use it as a general purpose set for every subject. To assess this, we chose the 5 most frequently selected channels across all subjects and cases and trained an algorithm on this reduced set of channels (like Fixed 64, this case only tuned the FDA regularization parameter). We call this case Fixed 5 and compared it to the Fixed 64, SWLDA, PCA-SWLDA, and MHPSO(4) cases. The channel set is shown in Figure 7 and the results are shown in Figure 8. A one-way repeated measures ANOVA with GG correction conducted on the single-trial accuracies of the 5 algorithms (factor: case) found a significant difference between cases (*ε* = 0.610, *F*(2.440,26.838) = 9.880, *P* < 0.001). After a* post hoc* Holm test, it was found that these differences were significant between pairs Fixed 64-SWLDA (*P* < 0.001) and MHPSO(4)-SWLDA (*P* < 0.001). Other significantly different pairs are Fixed 64-PCA-SWLDA (*P* = 0.012), Fixed 64-Fixed 5 (*P* = 0.032), and MHPSO(4)-PCA-SWLDA (*P* = 0.029). No significant differences were found in the 10 averaged-trial case. These results show that, although the algorithm using the full channel set provided the best accuracy, the Fixed 5 and MHPSO(4) algorithms yielded a performance comparable to or better than SWLDA and PCA-SWLDA using considerably fewer channels, thus being adequate for BMI systems where portability and simplicity are important. Although there was no significant difference between the Fixed 5 and MHPSO(4) cases (*P* = 0.197), there also seems to be a difference between using a fixed channel set assessed with MHPSO across all users or using a channel set adapted to each user. This difference, however, may become significant if the number of subjects is increased. Ultimately, the decision of whether to give priority to the accuracy or the compactness of the EEG channel set will depend on the particular constraints of each application. In the case that classification accuracy can be spared, the proposed algorithm can provide hints as to which channels can be used as general purpose set or to adapt a channel set to each particular user.

## 5. Conclusions

An algorithm based on FDA and MHPSO for the classification of P300 ERP signals was presented. The algorithm used MHPSO to find the FDA parameters and set of the fewest EEG channels that maximized the classification accuracy. The algorithm's performance was evaluated through offline analyses on datasets of auditory ERPs from sound discrimination experiments. The proposed method achieved a higher classification accuracy than that achieved by traditional methods while also using fewer channels. It was also found that it is possible to reduce the number of channels necessary for classification without greatly compromising the classification accuracy. Future work will be aimed at finding why, in addition to channels on the parietal and frontal regions typically associated to the P300 ERP, channels on the frontal polar region were selected. Also, given that the proposed algorithm can be easily extended to spatiotemporal feature selection, further research will focus on a version that tunes more binary variables to select the channel set and the time intervals within each channel that maximize performance. Lastly, future research will also explore the application of other swarm intelligence techniques such as Ant Colony Optimization and the Firefly Algorithm and compare it with the results obtained with MHPSO.
